# Supplementary material for: In Situ Fabrication of SnS2/SnO2 Heterostructures for Boosting Formaldehyde−Sensing Properties at Room Temperature
Source: Nanomaterials (Basel). 2023 Sep 4;13(17):2493. doi: 10.3390/nano13172493 (PMC10563078; doi:10.3390/nano13172493)
Supplement: Supplementary file 1 [file nanomaterials-13-02493-s001.zip › nanomaterials-2572111-supplementary.pdf]

nanomaterials

MDPI

Supporting information

In Situ Fabrication of SnS_2/SnO_2 Heterostructures for Boosting Formaldehyde−Sensing Properties at Room Temperature

Dan Meng [1], Zongsheng Xie [1], Mingyue Wang [2], Juhua Xu [3], Xiaoguang San [1,*], Jian Qi [4], Yue Zhang [1], Guosheng Wang [1] and Quan Jin [3,*]

[1] College of Chemical Engineering, Shenyang University of Chemical Technology, Shenyang 110142, China; mengdan0610@hotmail.com (D.M.); xzs19991015@163.com (Z.X.); meat−d@163.com (Y.Z.); m18240179987@163.com (G.W.)
[2] Institute for Superconducting and Electronic Materials (ISEM), Australian Institute for Innovative Materials (AIIM), Innovation Campus, University of Wollongong, Squires Way, North Wollongong, NSW 2500, Australia; mw663@uowmail.edu.au
[3] Key Laboratory of Automobile Materials (Ministry of Education), School of Materials Science and Engineering, Jilin University, Changchun 130022, China; hmm9911057@163.com
[4] State Key Laboratory of Biochemical Engineering, Institute of Process Engineering, Chinese Academy of Sciences, Beijing 100190, China; jqi@ipe.ac.cn
* Correspondence: sanxiaoguang@syuct.edu.cn (X.S.); qjin@jlu.edu.cn (Q.J.); Tel.: +86−24−8938−6342(D.M.)

1. Detailed synthesis process of SnS_2/SnO_2 composites with hollow spherical structures

The as-prepared SnO_2 hollow spheres (0.25 g) were dispersed in a mixed solvent of acetic acid (18 mL) and distilled water (2 mL) and then stirred vigorously for 30 min to obtain uniform SnO_2 dispersion. At the same time, the required amounts of thiourea (molar ratio: Sn: S=3:1, 3:2, and 3:5, respectively) and 20 mL of deionized water are added to a glass beaker and vigorously stirred for 30 min. After that, the obtained thiourea solution was added to the above SnO_2 dispersion and stirred at room temperature for 1 h. Subsequently, the mixed solution was transferred into a 50 mL Teflon-lined stainless steel autoclave and continuously reacted at 150°C for 2 h. After the reaction, the precipitate was collected by centrifugation, washed with distilled water and ethanol for 6 cycles, and dried at 60°C overnight to obtain SnS_2/SnO_2 composites. The final products were denoted as SnS_2/SnO_2-1, SnS_2/SnO_2-2, and SnS_2/SnO_2-3, respectively, with the molar ratio of Sn to S of 3:1, 3:2, and 3:5.

2. Detailed characterization

The crystalline structures of the SnS_2/SnO_2 composites were observed by X-ray diffraction (XRD, Shimadzu XRD-600 diffractometer) using Cu-Kα radiation (λ = 1.5418 Å) in 2θ range of 20° ~ 80°. The morphologies of products were examined by scanning electron microscope (SEM, ZEISS Ultra Plus) equipped with energy-dispersive X-ray photoelectron spectroscopy (EDS). More detailed characterization of the microstructures was further observed by high-resolution transmission electron microscopy (HRTEM) on a transmission electron microscopy system (TEM, JEOL, JEM-2100F). The chemical composition and electronic state of the obtained products were characterized by X-ray photoelectron spectroscopy (XPS, JEOL JPS9010MC). The specific surface area and pore size distribution were analyzed by N_2 adsorption/desorption isotherms (ASAP2020HD88) at 77 K. The semiconductor type of the products was analyzed by Mott–Schottky plots using an electrochemical workstation (MS, CHI660E, Shanghai, China) with a standard three-electrode configuration, wherein the electrolyte is 0.5 M Na_2SO_4 aqueous solution in the frequency of 1 kHz and scan rate of 10 mV/s. The electron paramagnetic resonance (EPR) spectra of samples obtained with a Bruke Emxplus spectrometer.

3. Theoretical limit of detection calculation:

The theoretical limit of detection (LOD) of the sensor can be calculated using the white signal noise (S_{noise}) generated during the gas test. LOD and S_{noise} have the following relationship:

$$LOD = 3\frac{S_{noise}}{b} \qquad (1)$$

Where b is the slope of the sensitivity linear fitting curves in Figure 8(b).

The S_{noise} can be obtained by calculating the root mean square deviation using the relative conductance change in the baseline. The calculation equation is as follows:

$$V_x^2 = \sum_{i=1}^{N}(Yi - Y)^2 \qquad (2)$$

$$S_{noise} = \sqrt{\frac{V_x^2}{N-1}} \qquad (3)$$

Where y_i is the measured data point, y is the average of the measured data points, and N is the number of selected data points.

4. BET analysis

The surface area and porosity of sensing materials are essential to improve the gas-sensing performance. N_2 adsorption-desorption test and BJH pore size distribution analysis were performed to confirm the specific surface area and porosity of SnO_2 hollow spheres and SnS_2/SnO_2-2 hollow spheres, as shown in Fig. S1. It can be observed that N_2 adsorption-desorption curves of two samples show type IV isotherm with H_3 hysteresis loop, indicating the characteristics of mesoporous-structures. The specific surface area of SnS_2/SnO_2--2 (92.5 m^2g^{-1}) is higher than that of SnO_2 (87.4 m^2g^{-1}), which indicates that SnS_2/SnO_2-2 hollow spheres can provide more gas adsorption active site compared to SnO_2 hollow spheres, thus improving the sensing performance. The corresponding pore size distribution of two samples (inset of Fig. S1) exhibits that most pores are concentrated at small pore distribution, mainly between 1~7 nm. There are also mesoporous and macroporous structures, which the gaps between pores or adjacent hollow spheres may cause. The above pores of samples acted as direct channels of diffusion for target gas molecules. They promoted rapid adsorption-desorption of gas molecules at the interface, improving the response-recover characteristics.

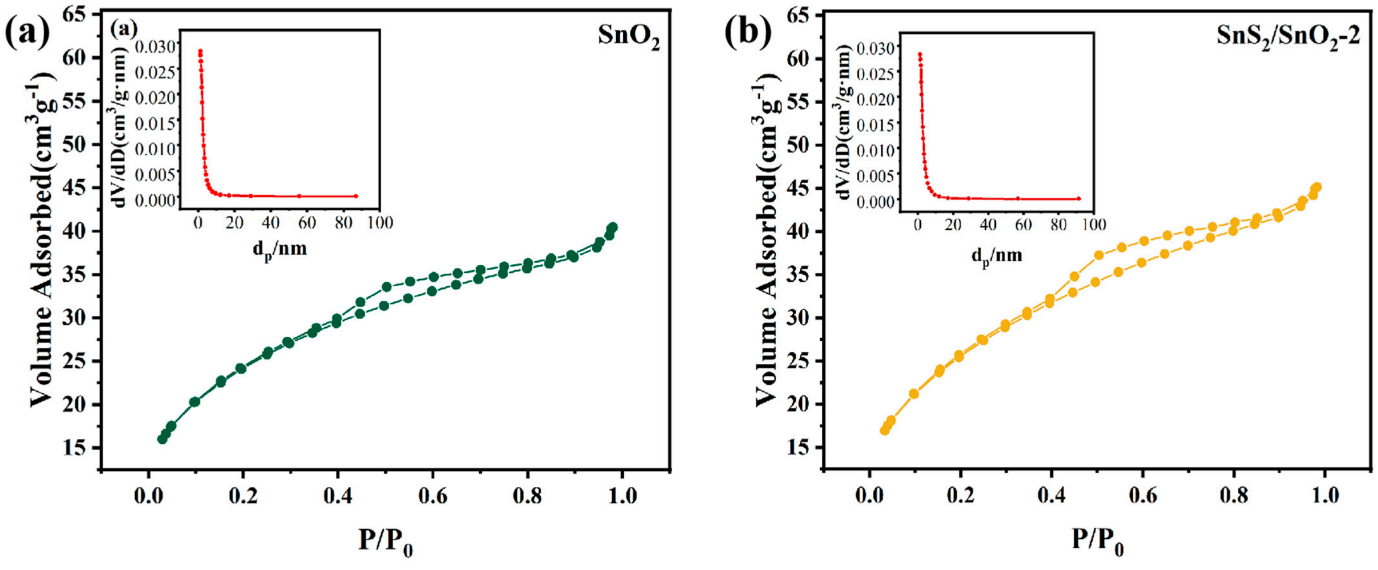

Figure S1. N_2 adsorption-desorption isotherms and corresponding BJH pore size distribution curves of (a) SnO_2 and (b) SnS_2/SnO_2-2 hollow spheres.

5. Theoretical calculation

All the density functional theory (DFT) calculations were performed in the Vienna ab initio simulation package (VASP). The Perdew-Burke-Ernzerhof (PBE) functional was used to describe electronic exchange and correlation. The projector-augmented wave method was employed to describe the interactions between the ion cores and valence electrons. In the calculation, plane wave truncation can be set to 500 eV to ensure the calculation accuracy, all atoms in the calculation are fully relaxed, and the accuracy of atomic convergence is set to 0.02 eV/A. The Brillouin zone was sampled using the Monkhorst-Pack scheme with a 2×2×1 k-point grid. The heterojunction model was constructed in this system by adding a 15 Å thick vacuum in the Z-direction to avoid interactions between adjacent layers. We used the DFT-D3 correction method to account for the van der Waals (vdW) interaction for our calculation. The adsorption energy (E_{ads}) was calculated as follows:

$$E_{ads} = E^*_{adsorbate} - E^* - E_{adsorbate}$$

$E^*_{adsorbate}$, E^*, and $E_{adsorbate}$ represented the total energy of adsorbed molecule and catalyst, the catalyst's energy, and the adsorbate's energy, respectively. A more negative E_{ads} value represented a more stable gas adsorption configuration.

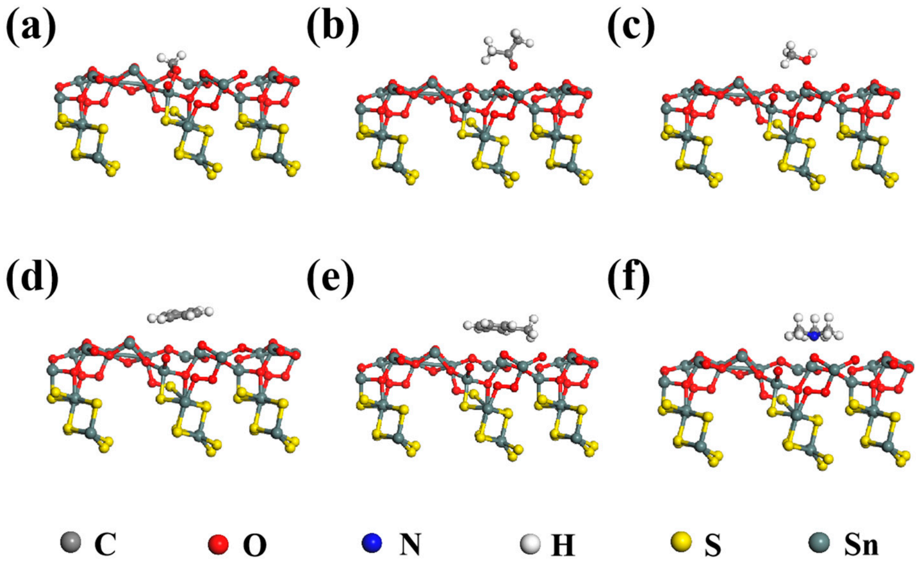

Figure S2. Optimized adsorption configurations of (a) formaldehyde, (b) ethanol, (c) methanol, (d) benzene, (e) toluene and (f) trimethylamine on the SnO_2/SnS_2 surface.
